# A Text-book of Human Physiology

**Published:** 1906-10

**Authors:** 


### BOOK REVIEWS.

A Text-book of Human Physiology. By Dr. Robert Tigerstedt, professor of physiology in the University of Helsingfers, Finland. Translated from the third German edition and edited by John R. Murlin, A.M., Ph.D., assistant professor of physiology in the University and Bellevue Hospital Medical College, New York City. With an introduction by Professor Graham Lusk, Ph.D., F.R.S. (Edinburg). Price, $4. Cloth. D. Appleton & Co., publishers, New York, N. Y.

Aside from the general excellence of this book, which may be taken for granted in view of the author's reputation as a physiologist, it presents a number of noteworthy and commendable features which differ from those of any other text-book yet published. The first of these is the manner of approach to the real subject. After a chapter on General Method, in which the author gives some instances of how exact physiological knowledge is gained, he takes the pains to lay a very broad foundation to the special subject of human physiology. The second chapter is a very compact one on the Physiology of the Cell. This constitutes the biological foundation. The next is a chapter on the Chemical Constituents of the Body which supplies the chemical foundation, but does not carry the reader to a needless length into the vast field of physiological chemistry. The fourth chapter is devoted to Metabolism and Nutrition, and in it the author points out (for the first time, we believe, in any physiology) that all the energy transformations of the body rest on the principle of the conservation of energy. He also describes the methods by which the very exact information of to-day regarding the general value of the foodstuffs, and the nutritive requirements of man under different circumstances is gained. This constitutes the physical basis of human physiology.

Another feature worthy of special mention is the classification of the subject-matter of the book. A clear and logical analysis underlies the order of presentation throughout. Following the introductory chapters just enumerated, the special subject of human physiology begins with a chapter on the blood.

The chapter on the circulation and the one on metabolism and nutrition fall within the author's own chosen fields of investigation. Needless to say, they contain much that is new and of great importance.

In his treatment of the physiology of the nervous system the author reaches the climax of the book, whether we consider it from the standpoint of the practitioner of medicine or of the general reader.

---

### NEWSPAPER NOTORIETY.

Consistency is often so rankly inconsistent that one flounders in an attempt to seek, by example, what is right. Almost daily on the front page of New York papers it is announced that Dr. William T. Bull performed an operation on one of the afflicted 400; Dr. Janeway "was in attendance"—or issued a bulletin, or some other equally well-known leader of the profession is brought before the public eye. Verily, to him that hath shall be given, and to him that hath not, even that which he hath shall be taken away. The question of newspaper notoriety is as much a matter of individual choice as the cut of one's hair or the style of coat he wears. If a man desires conspicuosity he wears his hair in a cascade over his shoulders, and long jim-swinger of stern and glossy blackness. If one chooses publicity through the press, he may have it abundantly, or he may totally avoid it, as he chooses.

In general it is stipulated as rather poor taste to be paraded professionally in the daily press, and most men shun it as they do long hair and frock coats. There are, however, extenuating circumstances which at times offer in vindication. But beware, ye lesser fry, thy name and deeds must be jealously guarded from the public eye, lest ye be placed among the goats; and let those who are strong beware how they lead the weak, for the pitfalls are many and the road is rough that leads to professional success.—*Editorial Southern Med. and Surg.*, August, 1906.

